# Editorial: Urban aging

**DOI:** 10.3389/fmed.2025.1738649

**Published:** 2025-11-24

**Authors:** Roberta Zupo, Fabio Castellana, Orazio Valerio Giannico, Francesco Desiante, Feliciana Catino, Rodolfo Sardone

**Affiliations:** 1 Department of Interdisciplinary Medicine (DIM), University of Bari Aldo Moro, Bari, Italy; 2 Urban Health Center-Local Health Authority of Taranto and the Municipality of Taranto, Taranto, Italy

**Keywords:** urban aging, built environment, healthy aging, migration, health equity, community-based care

## Introduction

The processes of urbanization and population aging are progressing in parallel, creating complex intersections between environmental design, health systems, and social structures. The Research Topic “*Urban aging”* brings together seven multidisciplinary studies that illuminate how cities can both challenge and sustain healthy aging. These contributions range from analyses of built environments and migration dynamics to novel care models and culturally embedded interventions. Collectively, they demonstrate that aging in cities depends not only on individual health behaviors but also on the fit between people, places, and policies.

## From environment to health: pathways and potentials

The first set of articles redefines the urban environment as an active determinant of wellbeing rather than a passive backdrop.

Yang et al. applied structural equation modeling to 10 urban communities to uncover the direct and indirect effects of the community built environment on older adults' health, as mediated by daily exercise. Their findings highlight that environmental design elements —walkability, safety, and accessible amenities—shape routine physical activity, which in turn mediates physical and mental health outcomes.

Similarly, Zhao et al. explored post-industrial cities, arguing that despite infrastructural decline and pollution, long-standing social networks and community identity can act as protective infrastructure. This reframes “healthy aging” beyond material resources to include the social fabric of place. Together, these studies advance a pathway-oriented view of urban health, suggesting that modifying environments can modify behaviors and, ultimately, aging trajectories.

## Mobility, institutions, and inequality

Migration and institutional structures also emerge as powerful determinants of aging in urban contexts.

Xu and Chen examined rural-to-urban migration among middle-aged and older adults using longitudinal data from the China Health and Retirement Longitudinal Study. Their analysis reveals that migrants reduced inpatient healthcare utilization after relocation, likely reflecting reduced reimbursement and weaker social integration.

At the city scale, Luo et al. analyzed spatial disparities of aging in Shenzhen through the lens of China's Hukou (household registration) system. Distinct spatial patterns emerged between registered residents and migrants—“west-to-east rising” among local Hukou holders and “south-high, north-low” among non-locals—underscoring institutionalized aging inequalities.

Pan et al. extended this analysis by exploring urban–rural cognitive disparities among empty-nest older adults. Using Blinder–Oaxaca decomposition, the authors found education to be the main driver of cognitive inequality, compounded by multimorbidity and depression.

Taken together, these studies reveal how institutional and structural determinants—registration status, education, chronic disease burden—shape the geography of aging as profoundly as physical infrastructure does.

## Care models, technology, and cultural integration

Aging well in cities also depends on accessible, trusted, and culturally congruent care.

Wan et al. employed a mixed-methods and machine-learning approach to identify the determinants of participation in shared elderly care models. E-health literacy and policy awareness emerged as primary drivers of engagement, alongside social belonging and technology usability. Their recommendations—digital literacy programs, inclusive design, and community-level communication—illustrate how behavioral engagement is socially and technologically mediated.

Complementing this, Geng et al. presented a randomized controlled trial protocol to assess the efficacy and safety of heat-sensitive moxibustion for the treatment of nocturia in older adults. Beyond its specific clinical scope, the protocol exemplifies the rigorous evaluation of culturally rooted therapies within evidence-based frameworks—bridging traditional medicine and modern geriatric care.

## Toward inclusive, place-based urban aging

Across diverse disciplines and methods, these seven studies converge on five actionable insights:

- Design for daily life. Built environments that facilitate safe, spontaneous activity yield cascading benefits for health and independence.
- Leverage community identity. Even in post-industrial or resource-poor settings, social cohesion and historical continuity can promote resilience.
- Integrate institutional reforms. Health equity requires portability of benefits and attention to administrative boundaries such as the Hukou system.
- Invest across the life course. Education and health literacy remain foundational to cognitive and functional health in later years.
- Co-produce care. Participation depends on usability, trust, and cultural fit—necessitating partnership between professionals, policymakers, and citizens.

## Future directions

The Research Topic “*Urban aging*” underscores the need for transdisciplinary frameworks that connect environmental design, social policy, and health science. Future research should pursue:

- Longitudinal causal designs linking urban interventions to behavioral and biological outcomes;
- Integration of mobility histories and institutional variables in aging datasets;
- Digital-health equity as an essential urban infrastructure;
- Implementation science to scale community-based models;
- Cross-cultural validation of interventions such as moxibustion within urban health systems.

## Concluding remarks

Aging in cities is not merely a demographic inevitability—it is also a design and policy opportunity. The seven contributions in *Urban Aging* collectively demonstrate that healthy longevity depends on the intersection of environment, equity, and engagement. By revealing how social capital, institutional design, and urban form interact, this Research Topic offers a roadmap for cities that aspire not only to accommodate aging populations but to enable thriving across generations.

